# Stem Cell-Derived Exosomes: A Potential Alternative Therapeutic Agent in Orthopaedics

**DOI:** 10.1155/2016/5802529

**Published:** 2016-01-19

**Authors:** John Burke, Ravindra Kolhe, Monte Hunter, Carlos Isales, Mark Hamrick, Sadanand Fulzele

**Affiliations:** ^1^Department of Orthopedics, Georgia Regents University, Augusta, GA 30912, USA; ^2^Department of Pathology, Georgia Regents University, Augusta, GA 30912, USA; ^3^Institute of Regenerative and Reparative Medicine, Georgia Regents University, Augusta, GA 30912, USA; ^4^Department of Cell Biology and Anatomy, Georgia Regents University, Augusta, GA 30912, USA

## Abstract

Within the field of regenerative medicine, many have sought to use stem cells as a promising way to heal human tissue; however, in the past few years, exosomes (packaged vesicles released from cells) have shown more exciting promise. Specifically, stem cell-derived exosomes have demonstrated great ability to provide therapeutical benefits. Exosomal products can include miRNA, other genetic products, proteins, and various factors. They are released from cells in a paracrine fashion in order to combat local cellular stress. Because of this, there are vast benefits that medicine can obtain from stem cell-derived exosomes. If exosomes could be extracted from stem cells in an efficient manner and packaged with particular regenerative products, then diseases such as rheumatoid arthritis, osteoarthritis, bone fractures, and other maladies could be treated with cell-free regenerative medicine via exosomes. Many advances must be made to get to this point, and the following review highlights the current advances of stem cell-derived exosomes with particular attention to regenerative medicine in orthopaedics.

## 1. Introduction

In the past few decades, regenerative medicine has sought to use human stem cells to heal human tissue. The use of mesenchymal stem cells (MSCs), induced pluripotent stem cells (iPSCs), and embryonic stem cells (ESCs) has shown promise in differentiation and proliferation in order to repair human tissue. Stem cells release a variety of products in a paracrine fashion that lead to their relevant effects. These products include growth factors, cytokines, and extracellular vesicles [1–3]. The extracellular vesicles secreted by cells are generally referred to as microvesicles, cell-derived vesicles, microparticles, shedding vesicles, and exosomes [2, 3]. The extracellular vesicles are classified on the basis of their cellular origin and/or biological function (Table 1) [2–5].

Exosomes are 40–100 nm diameter packaged vesicles containing specific proteins, lipids, factors, and/or genetic material that are secreted by multivesicular bodies upon stimulation [2, 3]. The numerous different types of materials contained within exosomes make them extremely promising in the field of regenerative medicine, and their lipid-bilayer membranes contain certain marker proteins that identify them specifically to particular cells [4]. Thus, exosomes are important players in cell-to-cell communication [5]. One particular facet of exosomes' packaging is that they contain lipid rafts, extremely concentrated areas of sphingolipids and cholesterol in the membrane that are important for cell communication and endocytosis. The presence of lipid rafts on exosomal membranes easily identifies their endosomal nature and can be used to detect exosomal presence instead of other vesicular products ([3, 5–8], see Table 1). A wide variety of materials can be transferred via exosomes, including specific proteins, RNA, and miRNA [9]. Furthermore, several studies have shown that horizontal transfer of mRNA and protein occurs through exosomal machinery, and the genetic material transferred successfully translated into the corresponding proteins [10–15]. Cantaluppi et al. demonstrated that microvesicles from endothelial progenitor cells fight kidney damage from ischemic events by packaging miRNA responsible for activating regenerative programs in the kidney [16]. These experiments demonstrate the promise of exosomes in regenerative medicine because if exosomes can be packaged in Good Manufacturing Practices (GMP), then exosomes can be utilized to transfer the corresponding proteins/genetic factors in order to combat disease.

There is a huge need for exosomes to be able to be packaged in GMP; however, at this time exosomes are not able to be manufactured in an easy, quick manner for use in clinical practices. Specific exosomes should be able to be purified, isolated, and cloned in order to be used in clinical settings. Exosomes need to be developed in a similar manner to interferon's (IFN) development into GMP. Interferon was known for its antiviral properties, but purifying it from a safe human source proved to be difficult until recently. This included finding a safe way to transfuse interferon to patients without causing adverse reactions, obtaining a safe and stable human source, and using recombinant technology to manufacture it in GMP [22]. While the heterogeneity and small content size of exosomes are very beneficial to their specific machinery, they make analyzing the small cargo extremely difficult. In order to manufacture viable exosomes, more extensive and efficient characterizations of exosomal cargo need to be employed than what currently exists. While stem cell therapies have been approved to treat cardiovascular disease, very few trials exist using exosomes; however, several preclinical studies are presently underway that are testing the abilities of exosomes to combat disease. One clinical trial includes the use of dendritic cell-derived exosomes which significantly augmented circulating NK cell numbers and NKG2D-dependent functions in the melanoma patients [23]. Another phase I clinical trial used ascites derived exosomes in combination with the granulocyte-macrophage colony-stimulating factor (GM-CSF) which induce beneficial tumor-specific antitumor cytotoxic T lymphocyte (CTL) response [24]. Projects such as these can increase our knowledge of how cancer progresses and provide potential therapeutics for treatment. Current knowledge of how miRNAs are selected and exported via exosomes must be better understood in order to utilize the miRNA machinery in regenerative and therapeutic mechanisms [16].

There is also new evidence that exosomes can be used to help identify disease pathogenesis. Because exosomes are released from cells in response to injury, it is to be expected that corresponding exosomal or other vesicular products would be released to combat the disease. For instance, juvenile arthritis patients demonstrate increased levels of exosomes released from macrophages that contain the nuclear protein DEK [25]. Recently, miRNAs trapped in exosome also show potential as biomarkers for the early cancer diagnosis. Tanaka and his coworkers reported elevated level of miR-21 in exosome isolated from esophageal squamous cell cancer patients' serum [15]. Exosome based miRNAs have great potential to be biomarker because miRNAs trapped in exosome are protected from RNase-dependent degradation and thus can be stably detected in body fluid such as plasma, serum, and urine.

## 2. Advantages of Exosome Based Therapy

Although there are many shortcomings with the current state of manufacturing exosomes, they have shown much promise and benefits at this time. Because of their physiochemical stability in the body as well as their multidimensional packaging, exosomes make great models for therapeutical medicine; stem cell-derived exosomes offer a method to provide cell-free regenerative medicine. Exosomes can be easily produced in the laboratory setting by treating stem cells under certain distress. The stem cells should release packaged exosomes in a paracrine fashion to combat the distress [17]. The exosomes released by the stem cells can be further analyzed in order to fully understand their contents. Exosomes are easily identifiable due to several markers such as size of about 40–100 nm [18] and their unique markers such as tetraspanins, flotillin, Alix, TSG101, and Rab5b ([17], see Table 1). Their specific makeup also provides exosomes with a cell-specific manner to dock and unload their cargo. This unique affinity for their cell target makes exosomes very potent mechanisms to transport proteins, miRNA, and so forth in the body without being degraded. The current state of knowledge of stem cell-derived exosomes is lacking for use in clinic, but it has shown high potential for future use in regenerative medicine.

## 3. Use of Exosomes for Regenerative Medicine in Orthopaedics

**Figure 1:**
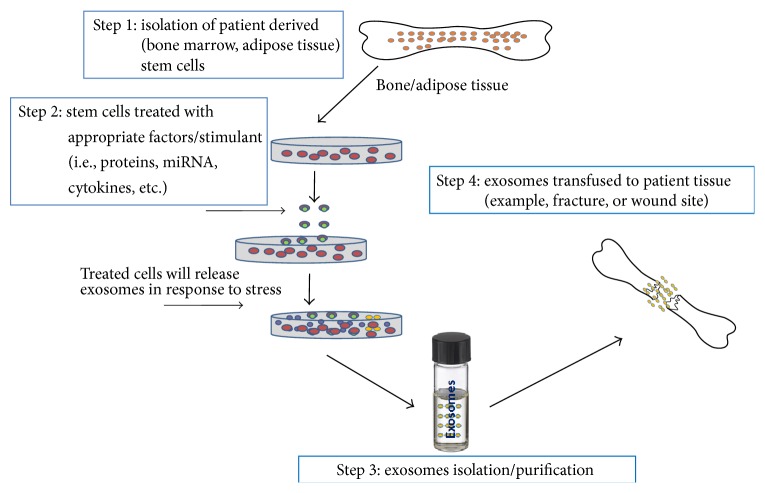
Schematic diagram of large scale exosome production and its use in tissue engineering.

Stem cell-derived exosomes have shown great promise in becoming a novel cell-free regenerative medicine (Figure 1). In one study, rats were subjected to middle cerebral artery occlusion and then treated with MSC-derived exosomes. These exosomes contained a significant amount of miRNA-133b which contributed to increased neurite branch numbers as well as total neurite length after middle cerebral artery occlusions [26]. Furthermore, there is increasing evidence that stem cell-derived exosomes as well as exosomes from other sources package specific miRNA to regulate cellular processes [27]. This offers an opportunity for exosome based therapeutics in musculoskeletal disorders because of the numerous known miRNAs that play vital roles in disease progression and prevention. If specific miRNAs can be proven to reduce inflammation/tissue damage in osteoarthritis (OA)/osteoporosis, then they could be packaged in exosomes to treat patients therapeutically in GMP. For example, one study found that silencing of miRNA-101 prevents cartilage degradation in mono-iodoacetate-induced arthritic rats [28]. Another experiment found that miRNA-140 and miRNA-455 are involved with cartilage development, and miRNA-9 and miRNA-98 are involved in endochondral ossification in bone matrix gelatin rat models [29]. These miRNAs along with many others have been identified and implicated in OA. These miRNAs can be packaged in exosomes for therapeutic use in osteoarthritis.

One such way to test these OA-associated miRNAs is to perform in vivo and in vitro studies with each miRNA and antisense miRNA. The antisense miRNA should have the opposite effect on cells as the regular miRNA does. This could provide the experimental basis to understand which miRNAs silence OA related pathways and which ones promote OA related pathways. For instance, human bone marrow-derived MSC exosomal miRNAs have been found to enrich the Wnt signaling pathway, leading to osteogenic differentiation [30]. The osteogenic differentiation included in the Wnt pathway includes osteoblast differentiation; this has the potential to be highly useful in osteoporosis and OA to combat degenerative changes.

It has been reported that human umbilical cord MSC-derived exosomes help in facilitating healing pathways in rat burn models [31]. The investigators also found that the wounds treated with exosomes exhibited accelerated reepithelialization and increased expression of CK19, PCNA, and collagen I in vivo. Wnt4 signaling plays a vital role in these processes. Further testing of the aforementioned miRNAs needs to be conducted in order to completely understand their specific involvement in OA and other diseases' pathways. Also, more research needs to be completed on how stem cells release and package their appropriate exosomal contents. One way to test how this occurs is to treat MSCs, ESCs, and iPSCs with OA inflammatory markers such as IL-6 and TNF-*α*. The theory is that when stem cells are exposed to inflammation and other degenerative markers, they should release exosomes to combat the inflammation and degradation (Figure 1). One study proved that dental pulp stem cells released exosomes that suppressed inflammation in mice [32]. If stem cells are introduced to the cellular products and conditions of bone fractures, OA, or osteoporosis-like conditions, then the stem cells should release particular exosomal machinery in response to promote healing and reverse degeneration (Figure 1). These exosomes should contain miRNAs, proteins, and other specific factors that would reverse the harmful conditions that the stem cells are exposed to. The exosomes released from stem cells should be identified by size as well as specific miRNAs and so forth that they contain. After exosomal cargo has been identified, the contents can be tested in in vivo and in vitro inflammatory and degenerative conditions of orthopaedic interest.

Raimondi and his group recently identified the crucial role of multiple myeloma-derived exosomes leading to osteoclast differentiation [33]. Multiple myeloma is the cancer of plasma cells and can lead to osteolytic lesions, hypercalcemia, bone pain, and bone degradation, as well as other factors involved with the presence of too many osteoclasts. In this study, multiple myeloma cells were found to release exosomes leading to differential proliferation of both murine and human osteoclasts. Preosteoclasts that were treated with these multiple myeloma-derived exosomes differentiated into osteoclasts. The results of this experiment have proven yet another manner of how exosomes significantly led to the cause of bone related complications. In OA, degeneration of the joint and articular cartilage occurs due to elevated level of inflammatory cytokines and MMPs (matrix metalloproteinases) in joints. Because we now know that these disease markers can occur through the products of exosomes, further experimentation must be conducted to see if these particular exosomes can be identified and prevented from being released. If stem cells were treated with MMP9, CTSK, and TRAP, then it is hypothesized that the SCs should release exosomes to combat the inflammation and prevent the progression of OA in vitro and in vivo. Such prevention could be accomplished through identifying a novel signaling pathway that leads to the release of proosteoclast driven signaling pathways. Particular miRNAs should be packaged within the exosomes that control the expression of osteoclast activity, which could be used to turn off such pathways in osteoporosis.

## 4. Exosomal Implications in Rheumatoid Arthritis (RA)

Perhaps one of the most promising uses of SC-derived exosomes within the field of orthopaedics involves rheumatoid arthritis (RA), an autoimmune disorder in which inflammation and T cells lead to pain and degradation of joints. Major Histocompatibility Complexes (MHCs) are involved in the development of RA, and Pêche et al. have demonstrated how exosomes can express MHCs [34]. One example of how exosomes can induce immune-regulated change is how tumor-derived exosomes produce antigen specific T cells in their targeted dendritic cells through MHCs, even though the dendritic cells had never been exposed to the tumor antigen [35–37]. Since MHCs are known to be formed and implicated in RA, further tests are required to see if and how exosomal machinery is involved. Furthermore, exosomes studied from synovial fibroblasts from individuals with RA have a membrane bound form of TNF-*α*, which leads to apoptotic resistance of T cells in RA [38]. Accordingly, the lack of apoptotic machinery for T cells in this experiment progresses the disease. If these harmful exosomes could be identified, then there could be an improvement in delaying the onset of RA. If the exosomes could be identified and packaged from a source of MSCs, then they could be used in therapeutical practices in order to help patients with RA. Citrullinated proteins are the source of the autoantigens in RA, and one study has shown that synovial exosomes contain these citrullinated proteins [39]. In order to fully understand exosomes' involvement in RA, these exosomes need to be more closely studied and identified in vivo. Rahman et al. have also shown that exosomes from MSCs can cause autoimmune responses in nonobese diabetic mice [40]. These MSCs secreted exosomes with autoantigens that activated B and T cell responses, once again implying exosomal presence in autoimmune disorders such as RA. While little has been done to find therapeutical exosomes for RA management, exosomes from human adipose MSCs have exhibited inhibitory effects on differentiation and activation of T cells [41]. These exosomes reduced T cell proliferation and reduced IFN-gamma release on in vitro cells. These findings are very important and relevant to all inflammatory related diseases, especially RA. Further research and development of new technologies/methods are required to be able to easily and quickly identify exosomes so that they can be used in therapeutical medicine.

## 5. Other Current Findings of Stem Cell-Derived Exosomes

Numerous potential therapeutic findings about SC-derived exosomes have been discovered within the past few years. Zhang et al. reported that exosomes released from human induced pluripotent stem cell-derived mesenchymal stem cells enhanced cutaneous wound healing in vitro and in animal models [42]. This occurred via the exosomes promoting collagen synthesis and angiogenesis. The animal models as well as in vitro experiments underwent faster reepithelialization, reduced scar widths, and promote collagen maturity. In another study, Shabbir et al. found that MSC-derived exosomes from normal donors as well as chronic wound patients led to increased proliferation and migration of fibroblasts in order to enhance wound healing [43]. These exosomes activated important wound healing signaling pathways such as Akt, ERK, and STAT3. They also induced the expression of various growth factors (HGF, IGF1, NGF, and SDF1). Furthermore, embryonic stem cell-derived exosomes have been proven to facilitate enhanced neovasvularization, cardiomyocyte survival, and reduced fibrosis in order to improve cardiac function in hearts following myocardial infarction [44]. These exosomes contained a significant enrichment of miRNA-290–295 within the ESC-derived exosomes in order to promote survival and proliferation within cardiomyocytes. MSC-derived exosomes have also been proven to accelerate skeletal muscle regeneration [45]. Nakamura and his group demonstrated that MSC-derived exosomes promoted myogenesis as well as angiogenesis via miRNAs including miRNA-494. This has profound implications within the field of orthopaedics. If these particular exosomes could be isolated from MSCs in GMP, then they could be used in therapeutical practice. Athletes and other patients who have damaged a hamstring muscle could use these exosomes to heal the muscle, leading to reduced costs of healthcare for the patient as well as a quicker recovery time. Since it is well documented that exosomes like these exist to repair tissue, then certainly some exosomes similar to these must exist to protect bones and joints. Multiple studies have already demonstrated the use of MSCs and miRNAs in bone and cartilage tissue engineering [46–48]. These findings suggest that SC-derived exosomes will one day be able to provide bone repair and regeneration therapy in clinical medicine. Since MSCs accomplish their regenerative tasks often through exosomes, further experiments need to be done in order to identify a novel exosome in vivo that can be used to repair cartilage in OA, RA, and other degenerative diseases.

## 6. Conclusive Remarks

The future of cell-free regenerative medicine is quickly evolving to become a reality for therapeutic use in patients, and it will occur because of the unique ability of stem cell-derived exosomes. These exosomes contain a variety of compounds such as miRNA, growth factors, and proteins to combat disease. Further experimentation must be done and more discoveries must be made in order for these SC-derived exosomes to become available for clinical use. If exosomes could be packaged with specific biological doses and certain products, then they could be used in clinic. While many new exciting discoveries have been made about SC-derived exosomes, there is much more to research to be able to use them therapeutically in clinic.

## Figures and Tables

**Table 1:** Classification of extracellular vesicles basis on their cellular origin.

Type of vesicle	Size	Origin	Markers	References
Exosomes	40–100 nm	Endosomes from many cell types	Tetraspanins, lipid rafts, flotillin, sphingomyelin, Alix, TSG101, Rab5b, and other endosomal related markers Most markers are not only specific to exosomes, however	[15, 17–19]

Microvesicles	20–1000 nm	Plasma membrane of many cell types	Wide variety of nonspecific makers including integrins, selectins, and CD40 ligand	[15, 17–20]

Membrane fragments	50–80 nm	Plasma membrane of epithelial cells	Prominin-1 (CD133)	[15, 20, 21]

Apoptotic bodies	1000–5000 nm	Plasma membrane from endoplasmic reticulum	Histones, DNA products, and phosphatidylserine	[15, 19, 20]
